# Supplementary material for: Evaluation of the Antimicrobial Capacity of Bacteria Isolated from Stingless Bee (Scaptotrigona aff. postica) Honey Cultivated in Açai (Euterpe oleracea) Monoculture
Source: Antibiotics (Basel). 2023 Jan 20;12(2):223. doi: 10.3390/antibiotics12020223 (PMC9952800; doi:10.3390/antibiotics12020223)
Supplement: Supplementary file 1 [file antibiotics-12-00223-s001.zip › antibiotics-2050887-supplementary.pdf]

Supplementary Data S1.Complete sequence of the bacterium SCA13. Result of the analysis of sequence similaritie s with the species Enterococcus faecalis in the BLAST database (Basic Local Alignment Search Tool - NCBI)

Query: None Query ID: lcl|Query_63917 Length: 962

>Enterococcus faecalis strain BE11 chromosome, complete genome
Sequence ID: CP110069.1 Length: 2913763
Range 1: 2865041 to 2865991

Score:1674 bits(906), Expect:0.0,
Identities:937/952(98%), Gaps:10/952(1%), Strand: Plus/Minus

Query 18 ATACATGCAAGTCGAACGCTTCTTTYCTCCCGAGTGCTTGCACTCAATTGGAAAGAGGAG 77
||||||||||||||||||||||||| ||||||||||||||||||||||||||||||||||
Sbjct 2865991 ATACATGCAAGTCGAACGCTTCTTTCCTCCCGAGTGCTTGCACTCAATTGGAAAGAGGAG 2865932

Query 78 TGGCGGACGGGTGAGTAACACGTGGGTAACCTACCCATCAGAGGGGGATAACACTTGGAA 137
||||||||||||||||||||||||||||||||||||||||||||||||||||||||||||
Sbjct 2865931 TGGCGGACGGGTGAGTAACACGTGGGTAACCTACCCATCAGAGGGGGATAACACTTGGAA 2865872

Query 138 ACAGGTGCTAATACCGCATAACAGTTTATGCCGCATGGCATAAGAGTGAAAGGCGCTTTC 197
||||||||||||||||||||||||||||||||||||||||||||||||||||||||||||
Sbjct 2865871 ACAGGTGCTAATACCGCATAACAGTTTATGCCGCATGGCATAAGAGTGAAAGGCGCTTTC 2865812

Query 198 GGGTGTCGCTGATGGATGGACCCGCGGTGCATTAGCTAGTTGGTGAGGTAACGGCTCACC 257
||||||||||||||||||||||||||||||||||||||||||||||||||||||||||||
Sbjct 2865811 GGGTGTCGCTGATGGATGGACCCGCGGTGCATTAGCTAGTTGGTGAGGTAACGGCTCACC 2865752

Query 258 AAGGCCRCGATGCATAGCCGACCTGAGAGGGTGATCGGCCACACTGGGACTGAGACACGG 317
|||||| |||||||||||||||||||||||||||||||||||||||||||||||||||||
Sbjct 2865751 AAGGCCACGATGCATAGCCGACCTGAGAGGGTGATCGGCCACACTGGGACTGAGACACGG 2865692

Query 318 CCCAGACTCCTACGGGAGGCAGCAGTAGGGAATCTTCGGCAATGGACGAAAGTCTGACCG 377
||||||||||||||||||||||||||||||||||||||||||||||||||||||||||||
Sbjct 2865691 CCCAGACTCCTACGGGAGGCAGCAGTAGGGAATCTTCGGCAATGGACGAAAGTCTGACCG 2865632

Query 378 AGCAACGCCGCGTGAGTGAAGAAGGTTTTCGGATCGTAAAACTCTGTTGTTAGAGAAGAA 437
||||||||||||||||||||||||||||||||||||||||||||||||||||||||||||
Sbjct 2865631 AGCAACGCCGCGTGAGTGAAGAAGGTTTTCGGATCGTAAAACTCTGTTGTTAGAGAAGAA 2865572

Query 438 CAAGGACGTTAGTAACTGAACGTCCCCTGACGGTATCTAACCAGAAAGCCACGGCTAAC

T 497
||||||||||||||||||||||||||||||||||||||||||||||||||||||||||||
Sbjct 2865571 CAAGGACGTTAGTAACTGAACGTCCCCTGACGGTATCTAACCAGAAAGCCACGGCTAA
CT 2865512

Query 498 ACGTGCCAGCAGCCGCGGTAATACGTAGGTGGCAAGCGTTGTCCGGATTTATTGGGCGT
A 557
||||||||||||||||||||||||||||||||||||||||||||||||||||||||||||
Sbjct 2865511 ACGTGCCAGCAGCCGCGGTAATACGTAGGTGGCAAGCGTTGTCCGGATTTATTGGGCG
TA 2865452

Query 558 AAGCGAGCGCAGGCGGTTTCTTAAGTCTGATGTGAAAGCCCCCGGCTCAACCGGGGAGG
G 617
||||||||||||||||||||||||||||||||||||||||||||||||||||||||||||
Sbjct 2865451 AAGCGAGCGCAGGCGGTTTCTTAAGTCTGATGTGAAAGCCCCCGGCTCAACCGGGGAG
GG 2865392

Query 618 TCATTGGAAACTGGGAGACTTGAGTGCAGAAGAGGAGAGTGGAATTCCATGTGTAGCGG
T 677
||||||||||||||||||||||||||||||||||||||||||||||||||||||||||||
Sbjct 2865391 TCATTGGAAACTGGGAGACTTGAGTGCAGAAGAGGAGAGTGGAATTCCATGTGTAGCG
GT 2865332

Query 678 GAAATGCGTAGATATATGGAGGAACACCAGTGGCGAAGGCGGCTCTCTGGTCTGTAACT
G 737
||||||||||||||||||||||||||||||||||||||||||||||||||||||||||||
Sbjct 2865331 GAAATGCGTAGATATATGGAGGAACACCAGTGGCGAAGGCGGCTCTCTGGTCTGTAAC
TG 2865272

Query 738 ACGCTGAGGCTCGAAAGCGTGGGGAGCAAACAGGATTAGATACCCTGGTAGTCCACGCC
G 797
||||||||||||||||||||||||||||||||||||||||||||||||||||||||||||
Sbjct 2865271 ACGCTGAGGCTCGAAAGCGTGGGGAGCAAACAGGATTAGATACCCTGGTAGTCCACGC
CG 2865212

Query 798 TAAACGATGAGTGCTAAGTGTTGGAGGGTTTCCGCCCTTCAGTGCTGCAGCAAACGCATT
857
||||||||||||||||||||||||||||||||||||||||||||||||||||||||||||
Sbjct 2865211 TAAACGATGAGTGCTAAGTGTTGGAGGGTTTCCGCCCTTCAGTGCTGCAGCAAACGCAT
T 2865152

Query 858 AAGCACTCCGCCTGGGGAGTACGAC-GCA-GGTTGAA-CTCAA-G-AA-TGACGGCCGC 9
11
||||||||||||||||||||||||| ||| ||||||| ||||| | || |||||| |||
Sbjct 2865151 AAGCACTCCGCCTGGGGAGTACGACCGCAAGGTTGAAACTCAAAGGAATTGACGGGGC
GC 2865092

Query 912 ACA-GCGGTGGAGCATGKG-TTT-ATTCGAAGCAACGCSGAARAACCTTACC 960
||| ||||||||||||| | ||| |||||||||||||| ||| |||||||||
Sbjct 2865091 ACAAGCGGTGGAGCATGTGGTTTAATTCGAAGCAACGC-GAAGAACCTTACC 2865041

Range 2: 222951 to 223904

```
Score:1664 bits(901), Expect:0.0,
Identities:938/955(98%),  Gaps:13/955(1%), Strand: Plus/Plus

Query  18      ATACATGCAAGTCGAACGCTTCTTTYCTCCCGAGTGCTTGCACTCAATTGGAAAGAGGAG
 77
               ||||||||||||||||||||||||| ||||||||||||||||||||||||||||||||||
Sbjct  222951  ATACATGCAAGTCGAACGCTTCTTTCCTCCCGAGTGCTTGCACTCAATTGGAAAGAGGA
G  223010

Query  78      TGGCGGACGGGTGAGTAACACGTGGGTAACCTACCCATCAGAGGGGGATAACACTTGGA
A  137
               ||||||||||||||||||||||||||||||||||||||||||||||||||||||||||||
Sbjct  223011  TGGCGGACGGGTGAGTAACACGTGGGTAACCTACCCATCAGAGGGGGATAACACTTGG
AA  223070

Query  138     ACAGGTGCTAATACCGCATAACAGTTTATGCCGCATGGCATAAGAGTGAAAGGCGCTTTC
  197
               ||||||||||||||||||||||||||||||||||||||||||||||||||||||||||||
Sbjct  223071  ACAGGTGCTAATACCGCATAACAGTTTATGCCGCATGGCATAAGAGTGAAAGGCGCTTT
C 223130

Query  198     GGGTGTCGCTGATGGATGGACCCGCGGTGCATTAGCTAGTTGGTGAGGTAACGGCTCACC
  257
               ||||||||||||||||||||||||||||||||||||||||||||||||||||||||||||
Sbjct  223131  GGGTGTCGCTGATGGATGGACCCGCGGTGCATTAGCTAGTTGGTGAGGTAACGGCTCAC
C 223190

Query  258     AAGGCCRCGATGCATAGCCGACCTGAGAGGGTGATCGGCCACACTGGGACTGAGACACG
G  317
               |||||| |||||||||||||||||||||||||||||||||||||||||||||||||||||
Sbjct  223191  AAGGCCACGATGCATAGCCGACCTGAGAGGGTGATCGGCCACACTGGGACTGAGACAC
GG  223250

Query  318     CCCAGACTCCTACGGGAGGCAGCAGTAGGGAATCTTCGGCAATGGACGAAAGTCTGACC
G  377
               ||||||||||||||||||||||||||||||||||||||||||||||||||||||||||||
Sbjct  223251  CCCAGACTCCTACGGGAGGCAGCAGTAGGGAATCTTCGGCAATGGACGAAAGTCTGACC
G  223310

Query  378     AGCAACGCCGCGTGAGTGAAGAAGGTTTTCGGATCGTAAAACTCTGTTGTTAGAGAAGA
A  437
               ||||||||||||||||||||||||||||||||||||||||||||||||||||||||||||
Sbjct  223311  AGCAACGCCGCGTGAGTGAAGAAGGTTTTCGGATCGTAAAACTCTGTTGTTAGAGAAGA
A  223370

Query  438     CAAGGACGTTAGTAACTGAACGTCCCCTGACGGTATCTAACCAGAAAGCCACGGCTAAC
T  497
               ||||||||||||||||||||||||||||||||||||||||||||||||||||||||||||
Sbjct  223371  CAAGGACGTTAGTAACTGAACGTCCCCTGACGGTATCTAACCAGAAAGCCACGGCTAAC
T  223430

Query  498     ACGTGCCAGCAGCCGCGGTAATACGTAGGTGGCAAGCGTTGTCCGGATTTATTGGGCGTA
  557
               ||||||||||||||||||||||||||||||||||||||||||||||||||||||||||||
```

```
Sbjct 223431  ACGTGCCAGCAGCCGCGGTAATACGTAGGTGGCAAGCGTTGTCCGGATTTATTGGGCGT
A  223490

Query  558     AAGCGAGCGCAGGCGGTTTCTTAAGTCTGATGTGAAAGCCCCCGGCTCAACCGGGGAGG
G  617
               ||||||||||||||||||||||||||||||||||||||||||||||||||||||||||||
Sbjct  223491  AAGCGAGCGCAGGCGGTTTCTTAAGTCTGATGTGAAAGCCCCCGGCTCAACCGGGGAGG
G  223550

Query  618     TCATTGGAAACTGGGAGACTTGAGTGCAGAAGAGGAGAGTGGAATTCCATGTGTAGCGG
T  677
               ||||||||||||||||||||||||||||||||||||||||||||||||||||||||||||
Sbjct  223551  TCATTGGAAACTGGGAGACTTGAGTGCAGAAGAGGAGAGTGGAATTCCATGTGTAGCGG
T  223610

Query  678     GAAATGCGTAGATATATGGAGGAACACCAGTGGCGAAGGCGGCTCTCTGGTCTGTAACT
G  737
               ||||||||||||||||||||||||||||||||||||||||||||||||||||||||||||
Sbjct  223611  GAAATGCGTAGATATATGGAGGAACACCAGTGGCGAAGGCGGCTCTCTGGTCTGTAACT
G  223670

Query  738     ACGCTGAGGCTCGAAAGCGTGGGGAGCAAACAGGATTAGATACCCTGGTAGTCCACGCC
G  797
               ||||||||||||||||||||||||||||||||||||||||||||||||||||||||||||
Sbjct  223671  ACGCTGAGGCTCGAAAGCGTGGGGAGCAAACAGGATTAGATACCCTGGTAGTCCACGCC
G  223730

Query  798     TAAACGATGAGTGCTAAGTGTTGGAGGGTTTCCGCCCTTCAGTGCTGCAGCAAACGCATT
 857
               ||||||||||||||||||||||||||||||||||||||||||||||||||||||||||||
Sbjct  223731  TAAACGATGAGTGCTAAGTGTTGGAGGGTTTCCGCCCTTCAGTGCTGCAGCAAACGCAT
T  223790

Query  858     AAGCACTCCGCCTGGGGAGTACGAC-GCA-GGTTGAA-CTCAA-G-AAT-GACGGG--CC 909
               ||||||||||||||||||||||||| ||| ||||||| ||||| | ||| ||||||  ||
Sbjct  223791  AAGCACTCCGCCTGGGGAGTACGACCGCAAGGTTGAAACTCAAAGGAATTGACGGGGG
CC 223850

Query  910     -GCAC-AGCGGTGGAGCATGKG-TTT-ATTCGAAGCAACGCSGAARAACCTTACC 960
                |||| |||||||||||||| | ||| ||||||||||||||  ||| |||||||||
Sbjct  223851  CGCACAAGCGGTGGAGCATGTGGTTTAATTCGAAGCAACGC-GAAGAACCTTACC  22390
4
```

Range 3: 961048 to 962001

Score:1664 bits(901), Expect:0.0,
Identities:938/955(98%), Gaps:13/955(1%), Strand: Plus/Plus

```
Query  18      ATACATGCAAGTCGAACGCTTCTTTYCTCCCGAGTGCTTGCACTCAATTGGAAAGAGGAG
 77
               ||||||||||||||||||||||||| ||||||||||||||||||||||||||||||||||
Sbjct  961048  ATACATGCAAGTCGAACGCTTCTTTCCTCCCGAGTGCTTGCACTCAATTGGAAAGAGGA
```

G 961107

Query 78 TGGCGGACGGGTGAGTAACACGTGGGTAACCTACCCATCAGAGGGGGATAACACTTGGA A 137

||||||||||||||||||||||||||||||||||||||||||||||||||||||||||||

Sbjct 961108 TGGCGGACGGGTGAGTAACACGTGGGTAACCTACCCATCAGAGGGGGATAACACTTGG AA 961167

Query 138 ACAGGTGCTAATACCGCATAACAGTTTATGCCGCATGGCATAAGAGTGAAAGGCGCTTTC 197

||||||||||||||||||||||||||||||||||||||||||||||||||||||||||||

Sbjct 961168 ACAGGTGCTAATACCGCATAACAGTTTATGCCGCATGGCATAAGAGTGAAAGGCGCTTT C 961227

Query 198 GGGTGTCGCTGATGGATGGACCCGCGGTGCATTAGCTAGTTGGTGAGGTAACGGCTCACC 257

||||||||||||||||||||||||||||||||||||||||||||||||||||||||||||

Sbjct 961228 GGGTGTCGCTGATGGATGGACCCGCGGTGCATTAGCTAGTTGGTGAGGTAACGGCTCAC C 961287

Query 258 AAGGCCRCGATGCATAGCCGACCTGAGAGGGTGATCGGCCACACTGGGACTGAGACACG G 317

|||||| |||||||||||||||||||||||||||||||||||||||||||||||||||||

Sbjct 961288 AAGGCCACGATGCATAGCCGACCTGAGAGGGTGATCGGCCACACTGGGACTGAGACAC GG 961347

Query 318 CCCAGACTCCTACGGGAGGCAGCAGTAGGGAATCTTCGGCAATGGACGAAAGTCTGACC G 377

||||||||||||||||||||||||||||||||||||||||||||||||||||||||||||

Sbjct 961348 CCCAGACTCCTACGGGAGGCAGCAGTAGGGAATCTTCGGCAATGGACGAAAGTCTGACC G 961407

Query 378 AGCAACGCCGCGTGAGTGAAGAAGGTTTTCGGATCGTAAAACTCTGTTGTTAGAGAAGA A 437

||||||||||||||||||||||||||||||||||||||||||||||||||||||||||||

Sbjct 961408 AGCAACGCCGCGTGAGTGAAGAAGGTTTTCGGATCGTAAAACTCTGTTGTTAGAGAAGA A 961467

Query 438 CAAGGACGTTAGTAACTGAACGTCCCCTGACGGTATCTAACCAGAAAGCCACGGCTAAC T 497

||||||||||||||||||||||||||||||||||||||||||||||||||||||||||||

Sbjct 961468 CAAGGACGTTAGTAACTGAACGTCCCCTGACGGTATCTAACCAGAAAGCCACGGCTAAC T 961527

Query 498 ACGTGCCAGCAGCCGCGGTAATACGTAGGTGGCAAGCGTTGTCCGGATTTATTGGGCGTA 557

||||||||||||||||||||||||||||||||||||||||||||||||||||||||||||

Sbjct 961528 ACGTGCCAGCAGCCGCGGTAATACGTAGGTGGCAAGCGTTGTCCGGATTTATTGGGCGT A 961587

Query 558 AAGCGAGCGCAGGCGGTTTCTTAAGTCTGATGTGAAAGCCCCCGGCTCAACCGGGGAGG G 617

||||||||||||||||||||||||||||||||||||||||||||||||||||||||||||

Sbjct 961588 AAGCGAGCGCAGGCGGTTTCTTAAGTCTGATGTGAAAGCCCCCGGCTCAACCGGGGAGG G 961647

```
Query  618     TCATTGGAAACTGGGAGACTTGAGTGCAGAAGAGGAGAGTGGAATTCCATGTGTAGCGG
T  677
            ||||||||||||||||||||||||||||||||||||||||||||||||||||||||||||
Sbjct  961648  TCATTGGAAACTGGGAGACTTGAGTGCAGAAGAGGAGAGTGGAATTCCATGTGTAGCGG
T  961707

Query  678     GAAATGCGTAGATATATGGAGGAACACCAGTGGCGAAGGCGGCTCTCTGGTCTGTAACT
G  737
            ||||||||||||||||||||||||||||||||||||||||||||||||||||||||||||
Sbjct  961708  GAAATGCGTAGATATATGGAGGAACACCAGTGGCGAAGGCGGCTCTCTGGTCTGTAACT
G  961767

Query  738     ACGCTGAGGCTCGAAAGCGTGGGGAGCAAACAGGATTAGATACCCTGGTAGTCCACGCC
G  797
            ||||||||||||||||||||||||||||||||||||||||||||||||||||||||||||
Sbjct  961768  ACGCTGAGGCTCGAAAGCGTGGGGAGCAAACAGGATTAGATACCCTGGTAGTCCACGCC
G  961827

Query  798     TAAACGATGAGTGCTAAGTGTTGGAGGGTTTCCGCCCTTCAGTGCTGCAGCAAACGCATT
 857
            ||||||||||||||||||||||||||||||||||||||||||||||||||||||||||||
Sbjct  961828  TAAACGATGAGTGCTAAGTGTTGGAGGGTTTCCGCCCTTCAGTGCTGCAGCAAACGCAT
T  961887

Query  858     AAGCACTCCGCCTGGGGAGTACGAC-GCA-GGTTGAA-CTCAA-G-AAT-GACGGG--CC 909
            ||||||||||||||||||||||||| ||| ||||||| ||||| | ||| ||||||  ||
Sbjct  961888  AAGCACTCCGCCTGGGGAGTACGACCGCAAGGTTGAAACTCAAAGGAATTGACGGGGG
CC 961947

Query  910     -GCAC-AGCGGTGGAGCATGKG-TTT-ATTCGAAGCAACGCSGAARAACCTTACC 960
             |||| |||||||||||||| | ||| |||||||||||||| ||| |||||||||
Sbjct  961948  CGCACAAGCGGTGGAGCATGTGGTTTAATTCGAAGCAACGC-GAAGAACCTTACC  96200
1
```

Range 4: 2417533 to 2418486

Score:1664 bits(901), Expect:0.0,
Identities:938/955(98%), Gaps:13/955(1%), Strand: Plus/Minus

```
Query  18      ATACATGCAAGTCGAACGCTTCTTTYCTCCCGAGTGCTTGCACTCAATTGGAAAGAGGAG
  77
             |||||||||||||||||||||||| |||||||||||||||||||||||||||||||||||
Sbjct  2418486  ATACATGCAAGTCGAACGCTTCTTTCCTCCCGAGTGCTTGCACTCAATTGGAAAGAGGA
G  2418427

Query  78      TGGCGGACGGGTGAGTAACACGTGGGTAACCTACCCATCAGAGGGGGATAACACTTGGA
A  137
             ||||||||||||||||||||||||||||||||||||||||||||||||||||||||||||
Sbjct  2418426  TGGCGGACGGGTGAGTAACACGTGGGTAACCTACCCATCAGAGGGGGATAACACTTGG
AA  2418367
```

Query 138 ACAGGTGCTAATACCGCATAACAGTTTATGCCGCATGGCATAAGAGTGAAAGGCGCTTT
C 197
||||||||||||||||||||||||||||||||||||||||||||||||||||||||||||
Sbjct 2418366 ACAGGTGCTAATACCGCATAACAGTTTATGCCGCATGGCATAAGAGTGAAAGGCGCTT
TC 2418307

Query 198 GGGTGTCGCTGATGGATGGACCCGCGGTGCATTAGCTAGTTGGTGAGGTAACGGCTCAC
C 257
||||||||||||||||||||||||||||||||||||||||||||||||||||||||||||
Sbjct 2418306 GGGTGTCGCTGATGGATGGACCCGCGGTGCATTAGCTAGTTGGTGAGGTAACGGCTCA
CC 2418247

Query 258 AAGGCCRCGATGCATAGCCGACCTGAGAGGGTGATCGGCCACACTGGGACTGAGACAC
GG 317
|||||| |||||||||||||||||||||||||||||||||||||||||||||||||||||
Sbjct 2418246 AAGGCCACGATGCATAGCCGACCTGAGAGGGTGATCGGCCACACTGGGACTGAGACAC
GG 2418187

Query 318 CCCAGACTCCTACGGGAGGCAGCAGTAGGGAATCTTCGGCAATGGACGAAAGTCTGACC
G 377
||||||||||||||||||||||||||||||||||||||||||||||||||||||||||||
Sbjct 2418186 CCCAGACTCCTACGGGAGGCAGCAGTAGGGAATCTTCGGCAATGGACGAAAGTCTGAC
CG 2418127

Query 378 AGCAACGCCGCGTGAGTGAAGAAGGTTTTCGGATCGTAAAACTCTGTTGTTAGAGAAGA
A 437
||||||||||||||||||||||||||||||||||||||||||||||||||||||||||||
Sbjct 2418126 AGCAACGCCGCGTGAGTGAAGAAGGTTTTCGGATCGTAAAACTCTGTTGTTAGAGAAG
AA 2418067

Query 438 CAAGGACGTTAGTAACTGAACGTCCCCTGACGGTATCTAACCAGAAAGCCACGGCTAAC
T 497
||||||||||||||||||||||||||||||||||||||||||||||||||||||||||||
Sbjct 2418066 CAAGGACGTTAGTAACTGAACGTCCCCTGACGGTATCTAACCAGAAAGCCACGGCTAA
CT 2418007

Query 498 ACGTGCCAGCAGCCGCGGTAATACGTAGGTGGCAAGCGTTGTCCGGATTTATTGGGCGT
A 557
||||||||||||||||||||||||||||||||||||||||||||||||||||||||||||
Sbjct 2418006 ACGTGCCAGCAGCCGCGGTAATACGTAGGTGGCAAGCGTTGTCCGGATTTATTGGGCG
TA 2417947

Query 558 AAGCGAGCGCAGGCGGTTTCTTAAGTCTGATGTGAAAGCCCCCGGCTCAACCGGGGAGG
G 617
||||||||||||||||||||||||||||||||||||||||||||||||||||||||||||
Sbjct 2417946 AAGCGAGCGCAGGCGGTTTCTTAAGTCTGATGTGAAAGCCCCCGGCTCAACCGGGGAG
GG 2417887

Query 618 TCATTGGAAACTGGGAGACTTGAGTGCAGAAGAGGAGAGTGGAATTCCATGTGTAGCGG
T 677
||||||||||||||||||||||||||||||||||||||||||||||||||||||||||||
Sbjct 2417886 TCATTGGAAACTGGGAGACTTGAGTGCAGAAGAGGAGAGTGGAATTCCATGTGTAGCG
GT 2417827

Query 678 GAAATGCGTAGATATATGGAGGAACACCAGTGGCGAAGGCGGCTCTCTGGTCTGTAACT

G 737

||||||||||||||||||||||||||||||||||||||||||||||||||||||||||||

Sbjct 2417826 GAAATGCGTAGATATATGGAGGAACACCAGTGGCGAAGGCGGCTCTCTGGTCTGTAAC
TG 2417767

Query 738 ACGCTGAGGCTCGAAAGCGTGGGGAGCAAACAGGATTAGATACCCTGGTAGTCCACGCC
G 797

||||||||||||||||||||||||||||||||||||||||||||||||||||||||||||

Sbjct 2417766 ACGCTGAGGCTCGAAAGCGTGGGGAGCAAACAGGATTAGATACCCTGGTAGTCCACGC
CG 2417707

Query 798 TAAACGATGAGTGCTAAGTGTTGGAGGGTTTCCGCCCTTCAGTGCTGCAGCAAACGCATT
857

||||||||||||||||||||||||||||||||||||||||||||||||||||||||||||

Sbjct 2417706 TAAACGATGAGTGCTAAGTGTTGGAGGGTTTCCGCCCTTCAGTGCTGCAGCAAACGCAT
T 2417647

Query 858 AAGCACTCCGCCTGGGGAGTACGAC-GCA-GGTTGAA-CTCAA-G-AAT-GACGGG--CC 909

||||||||||||||||||||||||| ||| ||||||| ||||| | ||| |||||| ||

Sbjct 2417646 AAGCACTCCGCCTGGGGAGTACGACCGCAAGGTTGAAACTCAAAGGAATTGACGGGGG
CC 2417587

Query 910 -GCAC-AGCGGTGGAGCATGKG-TTT-ATTCGAAGCAACGCSGAARAACCTTACC 960

 |||| |||||||||||||| | ||| |||||||||||||| ||| |||||||||

Sbjct 2417586 CGCACAAGCGGTGGAGCATGTGGTTTAATTCGAAGCAACGC-GAAGAACCTTACC 2417
533
